# Neuroimaging of pain in animal models: a review of recent literature

**DOI:** 10.1097/PR9.0000000000000732

**Published:** 2019-08-07

**Authors:** Joyce T. Da Silva, David A. Seminowicz

**Affiliations:** a Department of Neural and Pain Sciences, School of Dentistry, Center to Advance Chronic Pain Research, University of Maryland, Baltimore, MD, USA; b Department of Psychiatry, School of Medicine, Johns Hopkins University, Baltimore, USA

**Keywords:** Pain, Imaging, fMRI, MEMRI, PET, EEG, Animals

## Abstract

Neuroimaging of pain in animals allows us to better understand mechanisms of pain processing and modulation. In this review, we discuss recently published brain imaging studies in rats, mice, and monkeys, including functional magnetic resonance imaging (MRI), manganese-enhanced MRI, positron emission tomography, and electroencephalography. We provide an overview of innovations and limitations in neuroimaging techniques, as well as results of functional brain imaging studies of pain from January 1, 2016, to October 10, 2018. We then discuss how future investigations can address some bias and gaps in the field. Despite the limitations of neuroimaging techniques, the 28 studies reinforced that transition from acute to chronic pain entails considerable changes in brain function. Brain activations in acute pain were in areas more related to the sensory aspect of noxious stimulation, including primary somatosensory cortex, insula, cingulate cortex, thalamus, retrosplenial cortex, and periaqueductal gray. Pharmacological and nonpharmacological treatments modulated these brain regions in several pain models. On the other hand, in chronic pain models, brain activity was observed in regions commonly associated with emotion and motivation, including prefrontal cortex, anterior cingulate cortex, hippocampus, amygdala, basal ganglia, and nucleus accumbens. Neuroimaging of pain in animals holds great promise for advancing our knowledge of brain function and allowing us to expand human subject research. Additional research is needed to address effects of anesthesia, analysis approaches, sex bias and omission, and potential effects of development and aging.

## 1. Introduction

Brain imaging of pain involves the use of various techniques to either directly or indirectly image the structure, function, or pharmacology of the brain under painful conditions.^57,77^ These neuroimaging techniques include electroencephalography (EEG), multiple modalities of magnetic resonance imaging (MRI), and positron emission tomography (PET). Previous literature in human neuroimaging has shown that acute pain is usually processed by a main core of brain regions, whereas chronic pain, due to adaptive or maladaptive neuronal plasticity, modulates several brain circuits including somatosensory, cognitive, affective, motivational, and reward.^5,13,59,70^ Six recent animal functional MRI (fMRI) studies have contributed to the field through the advantages of using longitudinal investigations paired with pharmacological and nonpharmacological interventions.^17,40,46,61,75,88^ In addition, at the end of the imaging protocol, brain or other tissue of interest can be processed to further elucidate mechanisms and correlate changes in neuroimaging with molecular aspects of the tissue.

Previous reviews of animal neuroimaging studies of pain processing in animals have described activations in areas seen in common with human studies.^5,12,13,59,77,81^ The brain areas most commonly activated by noxious stimulation included the primary and secondary somatosensory cortices (S1, S2), insula, anterior cingulate cortex (ACC), and periaqueductal gray area (PAG).^5,12,13,59,77,81^ In addition, pharmacological imaging in animals also shows consistent activation patterns compared with humans with pain and opioid analgesia.^12,71,77^ This review will provide an overview of the current state of the field, as well as innovations and limitations in neuroimaging techniques. We searched for all articles published from January 1, 2016, to October 10, 2018, using the following search terms: animal, pain, brain, diffusion tensor imaging (DTI), EEG, functional MRI (fMRI), manganese-enhanced MRI (MEMRI), PET, and structural MRI. Exclusion criteria for this review were studies that did not include brain imaging and did not assess pain. In that period, 18 animal neuroimaging studies have used fMRI,^1–3,9,17,22,35,37,40,46,56,61,64,75,84,85,88,89^ whereas 10 other studies have used less commonly used techniques including 5 with MEMRI,^16,25,41,42,74^ 3 with PET,^19,44,78^ and 2 with EEG.^48,72^ There were no studies using diffusion tensor imaging or structural MRI in that period. Brain activity, connectivity, and receptor-binding capacity have been assessed in these studies. Finally, a brief discussion to encourage and aid future studies how investigate pain processing with neuroimaging tools is also explored.

## 2. Innovations and limitations in neuroimaging techniques

Neuroimaging studies using animals have investigated changes in brain mechanisms through different pain models over the last decade.^6,9,26,38,69^ We will discuss new approaches and limitations of each of the neuroimaging techniques used, considering the nature and features of the signal, as well as the setup and analysis methods (Table 1).

**Table 1:**
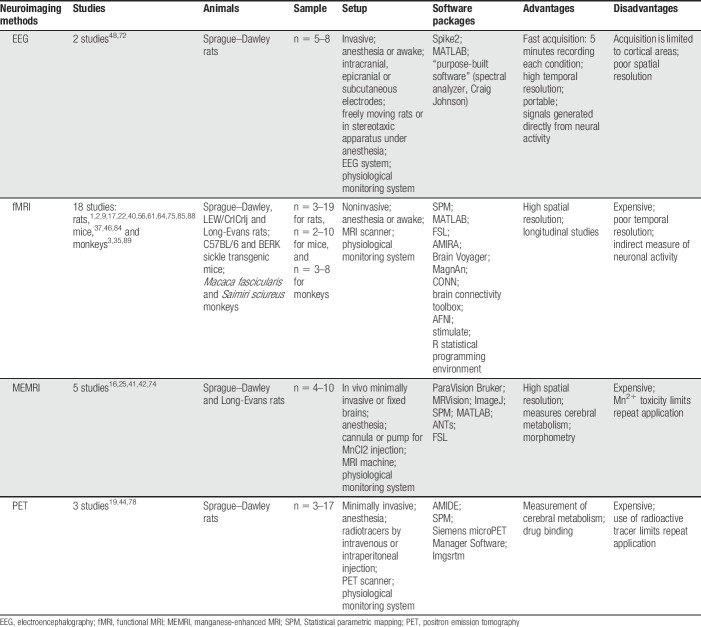
A summary of the features, advantages, and disadvantages of the neuroimaging methods discussed in this review.

### 2.1. Characteristics of the signal

The most commonly used brain imaging method in animals is MRI, which includes fMRI^1–3,9,17,22,35,37,40,46,56,61,64,75,84,85,88,89^ and MEMRI^16,25,41,42,74^ in the current literature of pain. Functional MRI usually uses the blood oxygenation level–dependent (BOLD) method as an indirect measure of neuronal activity associated with a task or stimulus, or using temporal interdependence of fMRI time series across regions to infer brain connectivity (see Refs. 4 and 80 for reviews). On the other hand, MEMRI uses manganese ions (Mn (2+)) as an MRI contrast agent that can be transported across active synapses and along axons (see Ref. 53 for review), revealing activation related to a task or stimulus across networks. Relatively fewer recent studies have used PET and EEG in pain neuroimaging, probably due to the disadvantages of these techniques, eg, radiotracer use and expense, and nonstandard setup for PET and EEG, respectively.^19,44,48,72,78^ Although PET and EEG have poor spatial resolution, they measure more direct neuronal activity than BOLD signal through glucose metabolism using fludeoxyglucose (FDG)-PET and brain electrical activity using EEG waveforms (see Refs. 18 and 65 for reviews). Efforts have been made to increase temporal and spatial resolution of neuroimaging techniques; however, there are specific limitations of each method (Table 1).

Spatial resolution refers to how accurately the measured activity is localized within the brain, and temporal resolution refers to how closely the measured activity corresponds to the timing of the actual neural activity.^45^ Functional MRI has a good spatial resolution (mm) that can be further improved using high magnetic field and scan duration (small animal scanners were 7 to over 11.7 Tesla (T) in recent studies,^1–3,17,22,35,40,46,56,61,64,75,84,85,88,89^ except 2 studies using 4.7 T^9,37^). However, some methods that increase signal-to-noise ratio can also come at a cost of greater distortion. For example, higher magnetic field strengths improve signal-to-noise ratio and contrast-to-noise ratio yielding higher resolution. Nevertheless, images with higher resolution are more sensitive to motion artifacts^36^ and the faster cardiac and respiratory rates from rodents.^43^ Furthermore, high-resolution demands long acquisition times, and motion artifacts can worsen with longer acquisition times.^27^ A good strategy is to image structures with enough detail and minimal distortion to achieve proper identification.^36^ The spatial resolution for in-plane resolution and slice thickness, respectively, in the reviewed fMRI articles ranged from 0.2 to 0.4 mm and 0.5 to 5 mm for mice studies, 0.2 to 0.8 mm and 0.5 to 2 mm for rat studies and 1 to 2 mm and 1 to 2.4 mm for monkey studies.^1–3,9,17,22,35,37,40,46,56,61,64,75,84,85,88,89^ By contrast, the temporal resolution of neuronal events is limited through the hemodynamic delay: typically, the BOLD response has a peak occurring ∼4–6 seconds after the onset of a brief neuronal event.^32^ One of the reviewed studies improved the temporal resolution by capturing more points of the hemodynamic response and interpolating those linearly (multishot EPI), which can also reduce the repetition time (TR) of magnetic pulses (500 ms).^85^ However, it is worth mentioning that those procedures cannot change the speed of the hemodynamic response. Manganese-enhanced MRI benefits from the use of T1-weighted images, providing better spatial resolution than fMRI, varying between 150 and 75 µm for rat studies,^16,25,41,42,74^ but the temporal resolution is up to 8 hours to achieve a good signal-to-noise ratio.^74^

The temporal resolution of PET is poor compared with fMRI and EEG, and is limited by the metabolism of the tracer molecule.^45^ Because of the low spatial resolution, the 3 reviewed PET studies reported the use of a structural MRI template to coregister the functional data to better localize the neuronal activity, which is a standard method in PET.^19,44,78^ Although spatial resolution is limited in PET, the method has the unique ability to measure glucose metabolism and receptor-binding levels, such as metabotropic glutamate receptor 5 (mGluR5) and opioid receptors (mu, delta, and kappa) in the whole brain.^19,44,78^ However, the temporal resolution of these phenomena is usually low due to the uptake time for the tracer to reach the brain and to achieve the peak of its radiation (minutes).

By contrast, EEG provides far better temporal resolution than other neuroimaging techniques, with capacity to record oscillatory activity in groups of neurons within milliseconds (∼3–500 Hz).^48,57^ We review only 2 EEG studies investigating pain in rats.^48,72^ They attempted to optimize the source localization using electrodes over the skull to assess primary somatosensory cortex, subcutaneous electrodes for whole-brain analysis, or craniotomy to implant a screw for prefrontal cortex (PFC) activity. However, the poor spatial resolution remains a challenge in EEG.^48,72^ Furthermore, the contribution of signals from subcortical structures to the EEG waveforms is debated.^47,66^

### 2.2. Setup and analysis

Some characteristics regarding setup for each technique are shown in Table 1. Briefly, fMRI in animals is performed by using an MRI scanner, radiofrequency coils, anesthesia, and a physiological monitoring system. Manganese-enhanced MRI requires the same setup as fMRI including the injection of the contrast agent. Practical issues are often faced in the MRI setup and require personnel training, including positioning of the animal and the receiver coil, choice of receiver coils, and monitoring of the anesthesia and physiological recordings. Once these issues are well settled by the research group, the quality of the data is highly improved. Positron emission tomography also uses a specific scanner, anesthesia, physiological monitoring system, and the injection of the radiotracer. By contrast, EEG requires more invasive procedures, such as surgery to place the EEG screws and electrodes in the head. The EEG setup essentially involves the use of electrodes, signal amplifier, and acquisition system. The 2 reviewed EEG articles were performed by different methods, which were recording from freely moving rats with intracranial and epicranial electrodes using tethered system^48^ and recording from anesthetized rats with subcutaneous electrodes and mechanical ventilation.^72^ Magnetic resonance imaging or PET scanners are usually part of a core facility and available for use at an hourly rate. On the other hand, EEG is frequently purchased by individual laboratories at a lower cost.

A potential confound of most neuroimaging studies is the use of anesthesia. Although fMRI can be performed in awake rats, the impact of stress and anxiety associated with the restraint in these studies is a potential confound.^9,17^ Although animals are habituated to the scanning environment, awake rodent fMRI protocols can cause long-lasting changes in physiological and brain responses to pain stimuli that are stress-related.^50^ Isoflurane has been the most common anesthetic source for fMRI,^1,3,22,37,40,56,61,75,84,85,89^ MEMRI,^25,41,42^ and PET.^19,44^ Halothane and sevoflurane are also inhalational anesthetics, but few studies have been used them because of the associated higher rate of liver injury and the decreased cerebral blood flow compared with isoflurane.^20,28,68,72,78,88^ Medetomidine is a sedative-analgesic that was injected by subcutaneous or intravenous infusion in 3 fMRI studies of pain.^2,46,64^ Although, medetomidine is gaining popularity as an anesthetic for rodent fMRI because it is also suitable for longitudinal studies, the BOLD signal changes during forepaw stimulation are similar to those observed under isoflurane, and it can induce bradycardia.^87^ Interestingly, a resting-state fMRI study has shown that combination of isoflurane and medetomidine at lower doses better resembled the connectivity pattern from awake rats compared with either isoflurane or medetomidine alone.^62^ The mechanism of this interaction is not yet understood but might be explained by the different actions of the anesthetics: isoflurane causes vasodilation and medetomidine causes vasoconstriction, which distinctively modulate the BOLD signal.^62,87^ Isoflurane–medetomidine and α-chloralose resembled the awake condition similarly,^62^ but α-chloralose is toxic and thus not suitable for longitudinal use, being used only in terminal preparation.^16^ Urethane was also administered mixed with α-chloralose in a MEMRI study with rats, but bradycardia, hyperventilation, and its carcinogenic nature are disadvantages of urethane.^29^ Anesthetics can induce several side effects; however, those are less relevant to signals from MEMRI and PET than to fMRI. BOLD relies on changes in the cerebral blood flow and oxygen level during the scan to capture the signal, whereas the MnCl_2_ contrast or the radiotracer is administered hours to days before the actual scan. An fMRI study with monkeys has used sedation through propofol.^35^ Propofol is an intravenous anesthetic that can reduce heart rate and cerebral blood flow, which may change the shape of the hemodynamic response function.^30^ Studies in rats and humans showed that propofol decreases connectivity in the thalamocortical and frontoparietal networks.^73,79^

An additional limitation in neuroimaging studies is the variety of analysis methods, which perpetuate the challenge of data reproducibility. Table 1 shows the diversity of software packages recently used in the reviewed articles. Software packages can differ in data input, preprocessing steps, sensitivity to correct motion, image manipulation tools, and statistical approaches and outputs.^10,55^ The most commonly used software packages for neuroimaging analysis are the Oxford Center for fMRI of the Brain (FMRIB) Software Library (FSL) and the statistical parametric mapping.^1–3,9,17,19,22,25,35,40,46,56,61,74,75,85,88,89^ Studies have shown that FSL and statistical parametric mapping perform similarly in fMRI statistical analysis.^55,63^ However, they have some weaknesses and strengths. Several studies have compared the performance of multiple software applications in human brain imaging,^23,39,55,63^ but no studies comparing the performance in animal neuroimaging studies have been published. Furthermore, it is worth mentioning that the preprocessing guidelines for animal data are far less established than for human data, eg, whereas human fMRI studies often apply spatial normalization to a common stereotaxic space, usually the Talairach or MNI templates, animal fMRI studies mostly report results in nonstandard space, which make direct comparisons and meta-analysis across studies unfeasible.^51^

The analysis of neuroimaging data can be performed using several approaches in software packages. The most common in fMRI studies of pain is the investigation of maps showing whole-brain activity from innocuous and noxious stimuli, which is basically created by modeling a block design paradigm of on and off conditions.^1–3,9,17,35,37,40,46,61,64,75,84,85,88,89^ Other methods have been used to assess specific regions and networks. Region of interest (ROI)-based analysis is the second most used method in recent fMRI studies of pain, which can measure the magnitude of the BOLD signal within ROIs and connectivity between an ROI and the whole brain or other ROIs during noxious stimulation or rest.^2,22,56,64,75,85,89^ Model-free independent component analysis was also used to identify networks related to pain processing.^1,9^ Independent component analysis establishes networks by regions that share the same response pattern without a previous assumption of ROIs.^15^ Graph theory,^37,46^ dynamic causal modeling, and band pass–filtered partial correlation analysis^75^ were used by few studies. These approaches can determine various characteristics of a network, such as the number of significant functional connections of an ROI,^37,46^ effective connectivity directly driven by response to stimuli,^76^ and exclusion of signal components directly time-locked to stimuli and shared among network regions.^75^

Functional connectivity was also inferred by MEMRI and EEG studies.^48,74^ Sperry et al.^74^ used a covariance pattern model to infer connectivity through the coactivated regions during pain using MEMRI. LeBlanc et al.^48^ investigated EEG by measuring the power transferred between brain regions suggesting functional connectivity. However, the localization of the brain areas was possible due to the invasive implanted screw electrodes, which could induce pain through infection or inflammation. Furthermore, 2 EEG studies of pain have investigated the different patterns of neuronal oscillations in the frequency domain, such as the power spectra^48,72^ and F50, which is the frequency below which 50% of the signal power is present and F95, which is the frequency below which 95% of the signal power is present.^72^ Results are discussed below.

Other challenges to be addressed in neuroimaging analysis are noise and statistical power. Noise decreases signal-to-noise ratio, which consequently lowers the power to detect signals related to the task of interest.^86^ Although most of the recent fMRI studies in animals have addressed this issue using physiological parameters, motion, and signals extracted from ventricles and white matter to regress out artifacts and increase signal,^3,9,17,22,37,40,46,56,75,89^ other neuroimaging techniques have not considered these variables in their current protocols.^16,19,25,41,42,44,48,72,74^ For example, non-neuronal physiological fluctuations due to pulsatility of blood flow in the brain and respiration can induce signal variance of the fMRI signal. These events are often comparable with that of the BOLD response during task or at rest, but several approaches are available to regress them out.^14^ From a statistical perspective, removal of non-BOLD artifacts improves effect size and statistical power. However, the easiest way of increasing statistical power is to increase sample size.^21^ Small sample sizes can produce highly variable estimates of the size and variance of the desired effect, which require the researches to carefully justify this factor in protocols.^58^ Sample size can be calculated by developed software packages, such as Neuro Power Tools (http://www.neuropowertools.org/).

## 3. Mechanisms proposed by recent neuroimaging studies of pain

### 3.1. Acute pain

**Figure 1.**
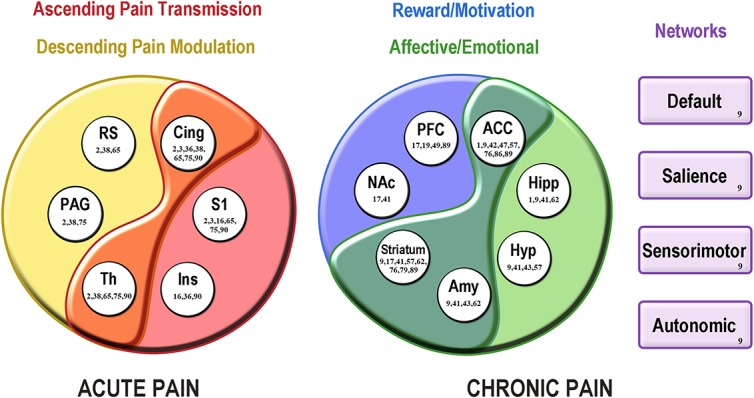
Altered brain activity and connectivity in animal models of acute and chronic pain. This figure summarizes the recent findings in neuroimaging studies of pain from January 1, 2016, to October 10, 2018, as indicated by the reference numbers. The color-coded circles show the proposed brain circuitries for acute and chronic pain. Acute pain is more associated with activation of regions from ascending and descending pain pathways, whereas chronic pain is more associated with regions related to reward/motivation and affective/emotional aspects of pain and altered functional connectivity of several networks. Overlap shows areas involved in both pathways or aspects of pain. ACC, anterior cingulate cortex; Amy, amygdala; Cing, cingulate cortex; Hipp, hippocampus; Hyp, hypothalamus; Ins, insula; NAc, nucleus accumbens; PAG, periaqueductal gray area; PFC, prefrontal cortex; RS, retrosplenial cortex; S1, primary somatosensory cortex; Th, thalamus.

Acute pain models were induced by incision, inflammation, or mechanical, thermal, or electrical stimulation and revealed a core pattern of nociception-evoked activations^2,3,16,25,35,37,64,72,74,89^ (Fig. 1). The most commonly activated regions during acute pain were S1, insula, cingulate cortex, thalamus, retrosplenial cortex, and PAG, all of which are part of ascending and descending nociceptive pathways.^2,3,16,25,35,37,64,89^ These findings are similar to those of human studies showing that S1, insula, cingulate cortex, and thalamus consistently respond to acute pain and are believed to play an important role in the sensory discriminative and affective aspects of pain processing.^57^ Although examining common regions identified across studies can be useful in identifying brain areas most associated with acute pain, individual studies can provide more detailed mechanisms. For example, Amirmohseni et al.^2^ examined differential activations in incisional and inflammatory pain using mechanical or electrical stimuli. The BOLD signal changes in cingulate cortex, thalamus, retrosplenial cortex, PAG, and striatum were significantly higher in rats with inflammatory pain than in rats with incisional pain or sham upon mechanical stimulation. Mechanical stimulation also produced a bilateral activation in S1, whereas electrical stimulation caused unilateral activation in S1 in both pain models. The presence of bilateral S1 activity after unilateral mechanical stimulation could result from uncrossed afferent pathways.^2^ Although the findings observed in knockout mice may not be directly correlated with results in physiologically normal animals, another fMRI study also confirmed that multisensory inputs are processed in different ways.^37^ They investigated the impact of a lack of the voltage-gated sodium channel Na_V_1.8 on brain structures and their interactions upon the perception of cold and heat noxious stimuli in knockout mice. BOLD signal amplitudes were strongly reduced in thalamus, cingulate cortex, and retrosplenial cortex upon noxious cold stimulation compared with heat stimulation.^37^ A reduced number of connections between affective and motivational-related regions were affected by the Na_V_1.8 knockout under cold but not under heat stimuli, eg, hippocampus and frontal cortex.^37^ These findings show the impact of Na_V_1.8 on noxious cold signaling and suggest its potential application in the treatment of especially cold pain states such as cold allodynia. Thus, it is important to carefully interpret the relationship between brain response and types of noxious stimulation to understand molecular mechanisms and target future treatment approaches.

Developmental study of pain processing has also been performed in rodents.^74^ In accordance with a recent study with human infants,^33^ Sperry et al.^74^ demonstrated that both sensory and affective-related regions are activated by pain in rat pups. Using MEMRI, the authors investigated brain activity after formalin injection in 12-day-old rat pups.^74^ The formalin injection induced enhanced activity of medulla, thalamus, sensory cortex, PFC, ACC, amygdala, and hypothalamus. Further research is needed to better understand the effects of development and aging on networks engaged in pain processing.

### 3.2. Chronic pain

Most recent animal neuroimaging studies have investigated brain mechanisms of chronic pain, including neuropathic pain, arthritis, fibromyalgia, migraine, visceral pain, trigeminal pain, and sickle cell.^1,9,17,19,25,40–42,44,46,48,56,61,75,78,84,85,88^ Despite the variety of pain models, types of stimulation, awake or anesthetic protocols, neuroimaging techniques, and analysis, a group of brain structures seems to be more involved in the later stages of pain (Fig. 1). A consistent observation is more widespread cerebral activity and connectivity in chronic pain compared with pain-free animals.^17,46,48,56,75,85,88^ Plastic changes have been primarily reported in S1 and ACC under chronic pain.^46,48,56,75,85,88^ An EEG study showed increased coherence between S1 and PFC at a late, but not early stage of neuropathic pain, suggesting that chronic pain increases connectivity between regions related to sensory/discriminative (S1) and negative/aversive (PFC) dimensions of pain.^48^ Prefrontal cortex has abnormal structure and function in humans with chronic pain, and studies have shown its potential as a therapeutic target for chronic pain conditions.^70^ Furthermore, increased PFC metabotropic glutamate receptor 5 (mGluR5) modulated negative mood symptoms of neuropathic pain, revealed by PET.^19^ Several studies using fMRI and MEMRI reported enhanced activity of PFC, ACC, hippocampus, amygdala, basal ganglia, and nucleus accumbens, but not the S1 in chronic pain.^1,17,41,42,46,56,61,75,85^ Allodynia-related brain activity was not dependent on S1 in rats with neuropathic pain and instead involved the nucleus accumbens and PFC.^17^ The participation of basal ganglia in chronic pain can also be demonstrated by the reduced opioid receptor availability in caudate-putamen of rats with chronic neuropathic pain.^78^ A PET study observed the positive correlation between anhedonia/depression-like behavior and the deficit of opioid receptor expression, suggesting the contribution of the opioid system to the comorbid depression in chronic pain.^78^ Regions mentioned above are known to be part of brain circuits related to emotion and motivation.^59^ Onishi et al.^61^ observed that activation of the amygdala and hippocampus could be, at least in part, responsible for the persistent electrical hyperalgesia and allodynia seen in a chronic stage of neuropathic pain. Similarly, Jeong and Kang^41^ demonstrated that neuropathic pain is transmitted to the S1 and parietal region through the cingulum bundle and limbic system. Furthermore, mechanical hyperalgesia from osteoarthritis pain induced more widespread brainstem and ACC activity, compared with stimulation of the noninjured paw.^1^ Thus, data from rodent studies reinforce the idea that chronic pain is associated with altered brain activity in many regions encoding affective, emotional, reward, and motivational contexts of pain.

Connectivity of the limbic system is increased in chronic pain and ACC may be a key region modulating this network.^46,56,75,85,88^ In addition to receiving afferent nociceptive information, ACC modulates the emotional and motivated behaviors of chronic pain through increased connectivity to striatum, hypothalamus, and mediodorsal thalamus.^46,56,75,88^ Morris et al.^56^ showed that higher ACC connectivity to hypothalamic/preoptic nuclei and the bed nucleus of the stria terminalis correlated with the reduction of motivated behavior, which was represented by burrowing behavior in rats with arthritis pain. Furthermore, allodynia induced by air-puff stimulation increased activity in the ACC and decreased activity in S1 in rats with chronic trigeminal pain.^75^ Anterior cingulate cortex also exerted an increased influence on superior colliculus and striatum, which suggests the role of ACC in the chronification of pain.^75^ Of the studies reviewed, one with migraine model reported altered resting-state fMRI functional connectivity in a number of networks previously identified in humans with the same chronic pain condition.^9,49^ The networks included the default mode, sensorimotor, interoceptive (salience), and autonomic circuits.^9^ The findings reviewed in this section showed similarities according to the chronic pain model; however, further research is needed to accurately replicate methods, estimate effect sizes, and compare them critically with the results of the original studies. Taken together, these findings suggest widespread modulation of emotional, motivational, and cognitive responses to chronic pain.

### 3.3. Descending pain modulation

Three studies reviewed the brain circuitry of endogenous pain inhibitory systems.^22,40,44^ In our own fMRI study, diffuse noxious inhibitory controls (DNIC) and brain networks were evaluated with respect to sex differences.^22^ We reported that the strength of DNIC behavior was higher in males compared with females and males without testosterone, and brain circuitry in the DNIC state is modulated by sex. Descending pain pathways, including connections between PAG, prelimbic cortex, ACC, and insula had stronger connectivity in males during DNIC induction. Females and nontestosterone males had increased connectivity in areas related to emotional and affective components of pain, such as ACC, hippocampus, and thalamus, compared with intact males. Nontestosterone males additionally had stronger connectivity of the nucleus accumbens with prelimbic cortex, ACC, insula, and thalamus. Our findings suggested that testosterone plays a key role in reinforcing the endogenous pain inhibitory system, while circuitries related to reward and emotion are more strongly recruited in the absence of testosterone.^22^ Human studies showed that females and males potentially differ in pain-related brain activation through different pathways, and testosterone may be a key factor in modulating pain sensitivity through descending circuits.^11,34,82,83^ We also previously investigated the estrogen stress–dependent interactions in response to noxious visceral stimulation in females using fMRI.^40^ Noxious visceral stimulation activated insula, ACC, and amygdala, whereas estrogen dramatically altered this visceral nociceptive processing in the brain after an acute stressor. These alterations included increased activation of amygdala, striatum-pallidum, nucleus accumbens, superior and inferior colliculi, and cerebellum, and decreased activation of the medial thalamus, hippocampus, and brainstem regions. These findings could reflect an upregulation of stress-related circuits and downregulation of descending modulatory circuits.^40^ The findings also support that sex hormones can dramatically alter nociceptive processing in the brain. The final reviewed study investigating descending pain pathways used PET to demonstrate that motor cortex stimulation (MCS) can attenuate neuropathic pain by increasing activity of striatum, thalamus, and cerebellum.^44^ The impact of acute and chronic pain on reward/motivation circuits gained from preclinical models is also found in the human literature.^60,90^ Furthermore, they performed immunohistochemistry to investigate changes in c-fos and serotonin expression, as well as extracellular electrophysiological recordings. These findings showed increased c-fos activity and amount of serotonin in lumbar levels of spinal cord and PAG, suggesting that MCS may be a descending modulator of neuropathic pain. Serotonin is a major neurotransmitter involved in descending pain modulation, as well as GABA and dopamine.^7^ Dopamine is known to be released by striatum, and MCS in neuropathic rats induced higher activity in this region.^44^ Thus, releases of dopamine by striatum and serotonin by PAG may be responsible for the pain relief achieved by MCS. Unfortunately, the sex of the rats was not reported.^44^

### 3.4. Interventions

Analgesic effects of pharmacological and alternative treatments have been evaluated using neuroimaging techniques.^35,41,44,48,64,72,88^ An EEG study investigated the effects of pregabalin (α2δ-ligand gabapentinoid from the class of anticonvulsants) and mexiteline (nonselective voltage-gated sodium channel blocker from the IB class of antiarrhythmic agents) on EEG power. They showed that pregabalin and mexiletine reversed the changes in EEG power of S1 and PFC and S1-PFC coherence in inflammatory and neuropathic pain models in rats, but similar effects were not observed when ibuprofen was given in an acute capsaicin model. Gabapentin, also an α2δ-ligand gabapentinoid, decreased activity in the posterior parietal association area, superior and inferior colliculi, S1, ACC, and cingulum bundle in rats with neuropathic pain who underwent MEMRI.^41^ Hama et al. also showed that pregabalin reduced bilateral ACC activation in monkeys with acute postoperative pain during non-noxious pressure stimulation using fMRI. However, morphine was able to reduce both ACC and bilateral insula activation under the same condition.^35^ This lack of reduced insula activation by pregabalin may represent a specific effect of this drug on ACC, while the morphine may have a broader action on pain-related brain responses. Changes in EEG responses to noxious thermal and electrical stimuli were seen after administration of 2 exogenous opioids, morphine, or opiorphin in rats.^72^ F50 and F95 values were similar to baseline after administration of either compound, which suggests that EEG signal may reflect the modulatory processes induced by opioids.^72^

Nonpharmacological therapies have also been investigated.^44,64,88^ Motor cortex stimulation improved neuropathic pain by modulating endogenous pain inhibitory system, as discussed above.^44^ An fMRI study showed that dorsal root ganglion electrical stimulation, ie, ganglionic field stimulation (GFS), could reduce activity of brain regions comprising the ascending spinothalamic system, specifically the contralateral thalamic nuclei, S1, and secondary somatosensory cortex (S2). BOLD responses in caudate-putamen, nucleus accumbens, globus pallidus, and amygdala were also reduced with GFS.^64^ Thus, GFS may not only reduce pain intensity but could also modulate the salience and motivational aspects of acute pain. Wu et al.^88^ have shown longitudinal changes in brain activity of rats with chronic neuropathic pain and electroacupuncture. They performed a sciatic nerve transection model followed by electroacupuncture 5 times per week for 4 months, and fMRI scans acquired every month after treatment. Areas of the somatosensory cortex were more activated in the first 2 months and then deactivated in the following 2 months in the treated group. By contrast, limbic areas were not constantly activated or deactivated, showing a fluctuating pattern in activity during treatment.^88^ All 3 of the above nonpharmacological treatments had analgesic effects. Taken together, these findings show that efficient pharmacological or nonpharmacological treatments can potentially reverse the maladaptive neuroplasticity of chronic and acute pain.

## 4. Considerations for future studies and conclusions

The neuroimaging studies discussed in this review aimed to determine brain functional responses to several models of acute and chronic pain. Each technique can be used to address a particular question. For example, fMRI is the gold standard for longitudinal studies because of its noninvasiveness; MEMRI uses the MnCl_2_ contrast, which is a calcium analog that can enter excitable cells and provide highly resolved functional and structural information; PET has a variety of radiotracer combinations that can be used to investigate cerebral metabolic processes, including receptor-binding and glucose metabolism; and EEG is the most direct technique to measure neuronal activity but is limited in spatial resolution. The use of multiple neuroimaging techniques can help address some limitations. Nonconventional anesthetic methods are encouraged, such as mixed isoflurane and medetomidine. Neuroimaging studies of awake animals might be limited by induced stress and unknown effects of the training. Subsequently, the variety of analytical methods is a common problem of neuroimaging techniques. It is recommended to use 2 or more software packages to enable higher reproducibility and refinement of the analytical methods in neuroimaging research.

Finally, investigators should take into consideration the problem of sex bias and omission in neuroimaging research of pain. From the 28 recent studies reviewed here, 20 were performed in males, 3 in females, 4 did not report the sex, and 1 was performed in males and females showing sex comparisons. Sex differences in pain perception have been shown in many studies in human subjects,^34,52,54,67^ and women are generally afflicted by chronic pain at higher rates than men.^8^ Furthermore, to the best of our knowledge, effects of aging have not been investigated by animal neuroimaging pain studies. Further research is needed to better understand the multidimensional aspects of pain in the brain. Several newer technologies could be combined with neuroimaging, including transcranial focused ultrasound, to induce neuromodulation in specific brain areas or opening of the blood–brain barrier^24^ and chemogenetic fMRI to remotely stimulate chemical-specific neurons during fMRI.^31^ To conclude, brain imaging studies in animals are adding important contributions to our understanding of pain processing, modulation, treatment effects, and treatment targets.

## Disclosures

The authors have no conflict of interest to declare.

## References

[1] Abaei M Sagar DR Stockley EG Spicer CH Prior M Chapman V Auer DP Neural correlates of hyperalgesia in the monosodium iodoacetate model of osteoarthritis pain. Mol Pain 2016;12:1–12. 10.1177/1744806916642445 PMC4956384 27068285

[2] Amirmohseni S Segelcke D Reichl S Wachsmuth L Görlich D Faber C Pogatzki-Zahn E Characterization of incisional and inflammatory pain in rats using functional tools of MRI. Neuroimage 2016;127:110–22. 26631818 10.1016/j.neuroimage.2015.11.052

[3] Asad AB Seah S Baumgartner R Feng D Jensen A Manigbas E Henry B Houghton A Evelhoch JL Derbyshire SW Chin CL Distinct BOLD fMRI responses of capsaicin-induced thermal sensation reveal pain-related brain activation in nonhuman primates. PLoS One 2016;11:e0156805. 27309348 10.1371/journal.pone.0156805 PMC4911046

[4] Bakshi R Advances in neuroimaging technology: state of the art. Rev Neurol Dis 2007;4:97–9. 17609642

[5] Baliki MN Apkarian AV Nociception, pain, negative moods, and behavior selection. Neuron 2015;87:474–91. 26247858 10.1016/j.neuron.2015.06.005 PMC4529956

[6] Baliki MN Chang PC Baria AT Centeno MV Apkarian AV Resting-sate functional reorganization of the rat limbic system following neuropathic injury. Sci Rep 2014;4:6186. 25178478 10.1038/srep06186 PMC4151103

[7] Bannister K Dickenson AH What do monoamines do in pain modulation? Curr Opin Support Palliat Care 2016;10:143–8. 27043287 10.1097/SPC.0000000000000207 PMC5604728

[8] Bartley EJ Fillingim RB Sex differences in pain: a brief review of clinical and experimental findings. Br J Anaesth 2013;111:52–8. 23794645 10.1093/bja/aet127 PMC3690315

[9] Becerra L Bishop J Barmettler G Kainz V Burstein R Borsook D Brain network alterations in the inflammatory soup animal model of migraine. Brain Res 2017;1:36–46. 10.1016/j.brainres.2017.02.001 PMC5731648 28167076

[10] Behroozi M Daliri MR Software tools for the analysis of functional magnetic resonance imaging. Basic Clin Neurosci 2012;3:71–83.

[11] Berman S Munakata J Naliboff BD Chang L Mandelkern M Silverman D Kovalik E Mayer EA Gender differences in regional brain response to visceral pressure in IBS patients. Eur J Pain 2000;4:157–72. 10957697 10.1053/eujp.2000.0167

[12] Borsook D Becerra L CNS animal fMRI in pain and analgesia. Neurosci Biobehav Rev 2011;35:1125–43. 21126534 10.1016/j.neubiorev.2010.11.005 PMC3076623

[13] Bushnell MC Ceko M Low LA Cognitive and emotional control of pain and its disruption in chronic pain. Nat Rev Neurosci 2013;14:502–11. 23719569 10.1038/nrn3516 PMC4465351

[14] Caballero-Gaudes C Reynolds RC Methods for cleaning the BOLD fMRI signal. Neuroimage 2017;154:128–49. 27956209 10.1016/j.neuroimage.2016.12.018 PMC5466511

[15] Calhoun VD Liu J Adali T A review of group ICA for fMRI data and ICA for joint inference of imaging, genetic, and ERP data. Neuroimage 2009;45(1 suppl):13. 10.1016/j.neuroimage.2008.10.057 PMC2651152 19059344

[16] Cha M Lee K Lee C Cho JH Cheong C Sohn JH Lee BH Manganese-enhanced MR imaging of brain activation evoked by noxious peripheral electrical stimulation. Neurosci Lett 2016;613:13–8. 26733299 10.1016/j.neulet.2015.11.027

[17] Chang PC Centeno MV Procissi D Baria A Apkarian AV Brain activity for tactile allodynia: a longitudinal awake rat functional magnetic resonance imaging study tracking emergence of neuropathic pain. PAIN 2017;158:488–97. 28135213 10.1097/j.pain.0000000000000788 PMC5303183

[18] Cherry SR Gambhir SS Use of positron emission tomography in animal research. ILAR J 2001;42:219–32. 11406721 10.1093/ilar.42.3.219

[19] Chung G Kim CY Yun YC Yoon SH Kim MH Kim YK Kim SJ Upregulation of prefrontal metabotropic glutamate receptor 5 mediates neuropathic pain and negative mood symptoms after spinal nerve injury in rats. Sci Rep 2017;7:017–09991. 10.1038/s41598-017-09991-8 PMC5575341 28851991

[20] Conzen PF Vollmar B Habazettl H Frink EJ Peter K Messmer K Systemic and regional hemodynamics of isoflurane and sevoflurane in rats. Anesth Analg 1992;74:79–88. 1734802 10.1213/00000539-199201000-00014

[21] Cremers HR Wager TD Yarkoni T The relation between statistical power and inference in fMRI. PLoS One 2017;12:e0184923. 29155843 10.1371/journal.pone.0184923 PMC5695788

[22] Da Silva JT Zhang Y Asgar J Ro JY Seminowicz DA Diffuse noxious inhibitory controls and brain networks are modulated in a testosterone-dependent manner in Sprague Dawley rats. Behav Brain Res 2018;349:91–7. 29733874 10.1016/j.bbr.2018.04.055 PMC7184319

[23] Dadar M Fonov VS Collins DL A comparison of publicly available linear MRI stereotaxic registration techniques. Neuroimage 2018;174:191–200. 29548850 10.1016/j.neuroimage.2018.03.025

[24] Deffieux T Demene C Pernot M Tanter M Functional ultrasound neuroimaging: a review of the preclinical and clinical state of the art. Curr Opin Neurobiol 2018;50:128–35. 29477979 10.1016/j.conb.2018.02.001

[25] Devonshire IM Burston JJ Xu L Lillywhite A Prior MJ Watson DJG Greenspon CM Iwabuchi SJ Auer DP Chapman V Manganese-enhanced magnetic resonance imaging depicts brain activity in models of acute and chronic pain: A new window to study experimental spontaneous pain? Neuroimage 2017;157:500–10. 28633971 10.1016/j.neuroimage.2017.06.034 PMC5607296

[26] Devonshire IM Greenspon CM Hathway GJ Developmental alterations in noxious-evoked EEG activity recorded from rat primary somatosensory cortex. Neuroscience 2015;305:343–50. 26254242 10.1016/j.neuroscience.2015.08.004

[27] Duyn JH The future of ultra-high field MRI and fMRI for study of the human brain. Neuroimage 2012;62:1241–8. 22063093 10.1016/j.neuroimage.2011.10.065 PMC3389184

[28] Fee JP Thompson GH Comparative tolerability profiles of the inhaled anaesthetics. Drug Saf 1997;16:157–70. 9098654 10.2165/00002018-199716030-00002

[29] Field KJ White WJ Lang CM Anaesthetic effects of chloral hydrate, pentobarbitone and urethane in adult male rats. Lab Anim 1993;27:258–69. 8366672 10.1258/002367793780745471

[30] Franceschini MA Radhakrishnan H Thakur K Wu W Ruvinskaya S Carp S Boas DA The effect of different anesthetics on neurovascular coupling. Neuroimage 2010;51:1367–77. 20350606 10.1016/j.neuroimage.2010.03.060 PMC2879067

[31] Giorgi A Migliarini S Galbusera A Maddaloni G Mereu M Margiani G Gritti M Landi S Trovato F Bertozzi SM Armirotti A Ratto GM De Luca MA Tonini R Gozzi A Pasqualetti M Brain-wide mapping of endogenous serotonergic transmission via chemogenetic fMRI. Cell Rep 2017;21:910–18. 29069598 10.1016/j.celrep.2017.09.087

[32] Glover GH Overview of functional magnetic resonance imaging. Neurosurg Clin N Am 2011;22:133–9; vii. 21435566 10.1016/j.nec.2010.11.001 PMC3073717

[33] Goksan S Baxter L Moultrie F Duff E Hathway G Hartley C Tracey I Slater R The influence of the descending pain modulatory system on infant pain-related brain activity. Elife 2018;7:37125. 10.7554/eLife.37125 PMC6133549 30201093

[34] Gupta A Mayer EA Fling C Labus JS Naliboff BD Hong JY Kilpatrick LA Sex-based differences in brain alterations across chronic pain conditions. J Neurosci Res 2017;95:604–16. 27870423 10.1002/jnr.23856 PMC5120652

[35] Hama A Natsume T Ogawa SY Awaga Y Hayashi I Matsuda A Takamatsu H Pain-related behavior and brain activation in a cynomolgus macaque model of postoperative pain. CNS Neurol Disord Drug Targets 2018;17:348–60. 29766827 10.2174/1871527317666180515121350

[36] Havsteen I Ohlhues A Madsen KH Nybing JD Christensen H Christensen A Are movement artifacts in magnetic resonance imaging a real problem?-a narrative review. Front Neurol 2017;8:232. 28611728 10.3389/fneur.2017.00232 PMC5447676

[37] Heindl-Erdmann C Zimmermann K Reeh P Brune K Hess A Activity and connectivity changes of central projection areas revealed by functional magnetic resonance imaging in NaV1.8-deficient mice upon cold signaling. Sci Rep 2017;7:017–00524. 10.1038/s41598-017-00524-x PMC5428718 28373680

[38] Hjornevik T Jacobsen LM Qu H Bjaalie JG Gjerstad J Willoch F Metabolic plasticity in the supraspinal pain modulating circuitry after noxious stimulus-induced spinal cord LTP. PAIN 2008;140:456–64. 19004552 10.1016/j.pain.2008.09.029

[39] Hoffmann M Carpenter TA Williams GB Sawiak SJ A survey of patient motion in disorders of consciousness and optimization of its retrospective correction. Magn Reson Imaging 2015;33:346–50. 25485789 10.1016/j.mri.2014.11.004

[40] Hubbard CS Karpowicz JM Furman AJ da Silva JT Seminowicz DA Traub RJ Estrogen-dependent visceral hypersensitivity following stress in rats: An fMRI study. Mol Pain 2016;12:1–10. 10.1177/1744806916654145 PMC4956385 27317579

[41] Jeong KY Kang JH Investigation of spinal nerve ligation-mediated functional activation of the rat brain using manganese-enhanced MRI. Exp Anim 2018;67:23–9. 28747592 10.1538/expanim.17-0033 PMC5814311

[42] Jeong KY Kim HM Kang JH Investigation of the functional difference between the pathological itching and neuropathic pain-induced rat brain using manganese-enhanced MRI. Acta Radiol 2016;57:861–8. 26385912 10.1177/0284185115604514

[43] Kalthoff D Seehafer JU Po C Wiedermann D Hoehn M Functional connectivity in the rat at 11.7T: Impact of physiological noise in resting state fMRI. Neuroimage 2011;54:2828–39. 20974263 10.1016/j.neuroimage.2010.10.053

[44] Kim J Ryu SB Lee SE Shin J Jung HH Kim SJ Kim KH Chang JW Motor cortex stimulation and neuropathic pain: how does motor cortex stimulation affect pain-signaling pathways? J Neurosurg 2016;124:866–76. 26274988 10.3171/2015.1.JNS14891

[45] Kimberley TJ Lewis SM Understanding neuroimaging. Phys Ther 2007;87:670–83. 17429004 10.2522/ptj.20060149

[46] Komaki Y Hikishima K Shibata S Konomi T Seki F Yamada M Miyasaka N Fujiyoshi K Okano HJ Nakamura M Okano H Functional brain mapping using specific sensory-circuit stimulation and a theoretical graph network analysis in mice with neuropathic allodynia. Sci Rep 2016;6:37802. 27898057 10.1038/srep37802 PMC5127182

[47] Krishnaswamy P Obregon-Henao G Ahveninen J Khan S Babadi B Iglesias JE Hämäläinen MS Purdon PL Sparsity enables estimation of both subcortical and cortical activity from MEG and EEG. Proc Natl Acad Sci U S A 2017;114:E10465–74. 29138310 10.1073/pnas.1705414114 PMC5715738

[48] LeBlanc BW Bowary PM Chao YC Lii TR Saab CY Electroencephalographic signatures of pain and analgesia in rats. PAIN 2016;157:2330–40. 27347647 10.1097/j.pain.0000000000000652

[49] Liu J Zhao L Lei F Zhang Y Yuan K Gong Q Liang F Tian J Disrupted resting-state functional connectivity and its changing trend in migraine suffers. Hum Brain Mapp 2015;36:1892–907. 25640857 10.1002/hbm.22744 PMC6869678

[50] Low LA Bauer LC Pitcher MH Bushnell MC Restraint training for awake functional brain scanning of rodents can cause long-lasting changes in pain and stress responses. PAIN 2016;157:1761–72. 27058679 10.1097/j.pain.0000000000000579 PMC4949008

[51] Mandal PK Mahajan R Dinov ID Structural brain atlases: design, rationale, and applications in normal and pathological cohorts. J Alzheimers Dis 2012;31(suppl 3):S169–188. 22647262 10.3233/JAD-2012-120412 PMC4324755

[52] Martin LJ Acland EL Cho C Gandhi W Chen D Corley E Kadoura B Levy T Mirali S Tohyama S Khan S MacIntyre LC Carlson EN Schweinhardt P Mogil JS Male-Specific Conditioned pain hypersensitivity in mice and humans. Curr Biol 2019;29:192–e4. 30639112 10.1016/j.cub.2018.11.030

[53] Massaad CA Pautler RG Manganese-enhanced magnetic resonance imaging (MEMRI). Methods Mol Biol 2011;711:145–74. 21279601 10.1007/978-1-61737-992-5_7 PMC3285478

[54] Mogil JS Sex differences in pain and pain inhibition: multiple explanations of a controversial phenomenon. Nat Rev Neurosci 2012;13:859–66. 23165262 10.1038/nrn3360

[55] Morgan VL Dawant BM Li Y Pickens DR Comparison of fMRI statistical software packages and strategies for analysis of images containing random and stimulus-correlated motion. Comput Med Imaging Graph 2007;31:436–46. 17574816 10.1016/j.compmedimag.2007.04.002 PMC2570159

[56] Morris LS Sprenger C Koda K de la Mora DM Yamada T Mano H Kashiwagi Y Yoshioka Y Morioka Y Seymour B Anterior cingulate cortex connectivity is associated with suppression of behaviour in a rat model of chronic pain. Brain Neurosci Adv 2018;2:2398212818779646. 30246156 10.1177/2398212818779646 PMC6109941

[57] Morton DL Sandhu JS Jones AK Brain imaging of pain: state of the art. J Pain Res 2016;9:613–24. 27660488 10.2147/JPR.S60433 PMC5019436

[58] Mumford JA A power calculation guide for fMRI studies. Soc Cogn Affect Neurosci 2012;7:738–42. 22641837 10.1093/scan/nss059 PMC3427872

[59] Navratilova E Morimura K Xie JY Atcherley CW Ossipov MH Porreca F Positive emotions and brain reward circuits in chronic pain. J Comp Neurol 2016;524:1646–52. 26788716 10.1002/cne.23968 PMC4809757

[60] Navratilova E Porreca F Reward and motivation in pain and pain relief. Nat Neurosci 2014;17:1304–12. 25254980 10.1038/nn.3811 PMC4301417

[61] Onishi O Ikoma K Oda R Yamazaki T Fujiwara H Yamada S Tanaka M Kubo T Sequential variation in brain functional magnetic resonance imaging after peripheral nerve injury: A rat study. Neurosci Lett 2018;673:150–6. 29524643 10.1016/j.neulet.2018.03.003

[62] Paasonen J Stenroos P Salo RA Kiviniemi V Gröhn O Functional connectivity under six anesthesia protocols and the awake condition in rat brain. Neuroimage 2018;172:9–20. 29414498 10.1016/j.neuroimage.2018.01.014

[63] Pauli R Bowring A Reynolds R Chen G Nichols TE Maumet C Exploring fMRI results space: 31 variants of an fMRI Analysis in AFNI, FSL, and SPM. Front Neuroinform 2016;10:24. 27458367 10.3389/fninf.2016.00024 PMC4932120

[64] Pawela CP Kramer JM Hogan QH Dorsal root ganglion stimulation attenuates the BOLD signal response to noxious sensory input in specific brain regions: Insights into a possible mechanism for analgesia. Neuroimage 2017;147:10–18. 27876655 10.1016/j.neuroimage.2016.11.046

[65] Ploner M Sorg C Gross J Brain rhythms of pain. Trends Cogn Sci 2017;21:100–10. 28025007 10.1016/j.tics.2016.12.001 PMC5374269

[66] Portnova GV Tetereva A Balaev V Atanov M Skiteva L Ushakov V Ivanitsky A Martynova O Correlation of BOLD signal with linear and nonlinear patterns of EEG in resting state EEG-informed fMRI. Front Hum Neurosci 2018;11:654. 29375349 10.3389/fnhum.2017.00654 PMC5767270

[67] Rosen S Ham B Mogil JS Sex differences in neuroimmunity and pain. J Neurosci Res 2017;95:500–8. 27870397 10.1002/jnr.23831

[68] Safari S Motavaf M Seyed Siamdoust SA Alavian SM Hepatotoxicity of halogenated inhalational anesthetics. Iran Red Crescent Med J 2014;16:e20153. 25593732 10.5812/ircmj.20153 PMC4270648

[69] Seminowicz DA Jiang L Ji Y Xu S Gullapalli RP Masri R Thalamocortical asynchrony in conditions of spinal cord injury pain in rats. J Neurosci 2012;32:15843–8. 23136423 10.1523/JNEUROSCI.2927-12.2012 PMC3500510

[70] Seminowicz DA Moayedi M The dorsolateral prefrontal cortex in acute and chronic pain. J Pain 2017;18:1027–35. 28400293 10.1016/j.jpain.2017.03.008 PMC5581265

[71] Shah YB Haynes L Prior MJ Marsden CA Morris PG Chapman V Functional magnetic resonance imaging studies of opioid receptor-mediated modulation of noxious-evoked BOLD contrast in rats. Psychopharmacology (Berl) 2005;180:761–73. 15778889 10.1007/s00213-005-2214-6

[72] Singh P Kongara K Harding D Ward N Dukkipati VSR Johnson C Chambers P Comparison of electroencephalographic changes in response to acute electrical and thermal stimuli with the tail flick and hot plate test in rats administered with opiorphin. BMC Neurol 2018;18:43. 29673329 10.1186/s12883-018-1047-y PMC5907193

[73] Song XX Yu BW Anesthetic effects of propofol in the healthy human brain: functional imaging evidence. J Anesth 2015;29:279–88. 25056258 10.1007/s00540-014-1889-4

[74] Sperry MM Kandel BM Wehrli S Bass KN Das SR Dhillon PS Gee JC Barr GA Mapping of pain circuitry in early post-natal development using manganese-enhanced MRI in rats. Neuroscience 2017;352:180–9. 28391012 10.1016/j.neuroscience.2017.03.052 PMC7276061

[75] Spisák T Pozsgay Z Aranyi C Dávid S Kocsis P Nyitrai G Gajári D Emri M Czurkó A Kincses ZT Central sensitization-related changes of effective and functional connectivity in the rat inflammatory trigeminal pain model. Neuroscience 2017;344:133–47. 28003158 10.1016/j.neuroscience.2016.12.018

[76] Stephan KE Friston KJ Analyzing effective connectivity with functional magnetic resonance imaging. Wiley Interdiscip Rev Cogn Sci 2010;1:446–59. 21209846 10.1002/wcs.58 PMC3013343

[77] Thompson SJ Bushnell MC Rodent functional and anatomical imaging of pain. Neurosci Lett 2012;520:131–9. 22445887 10.1016/j.neulet.2012.03.015

[78] Thompson SJ Pitcher MH Stone LS Tarum F Niu G Chen X Kiesewetter DO Schweinhardt P Bushnell MC Chronic neuropathic pain reduces opioid receptor availability with associated anhedonia in rat. PAIN 2018;159:1856–66. 29794614 10.1097/j.pain.0000000000001282 PMC6095806

[79] Tu Y Yu T Fu XY Xie P Lu S Huang XQ Gong QY Altered thalamocortical functional connectivity by propofol anesthesia in rats. Pharmacology 2011;88:322–6. 22116025 10.1159/000334168

[80] van den Heuvel MP Hulshoff Pol HE Exploring the brain network: a review on resting-state fMRI functional connectivity. Eur Neuropsychopharmacol 2010;20:519–34. 20471808 10.1016/j.euroneuro.2010.03.008

[81] Vesce G Micieli F Chiavaccini L Preclinical imaging anesthesia in rodents. Q J Nucl Med Mol Imaging 2017;61:1–18. 27858407 10.23736/S1824-4785.16.02951-4

[82] Vincent K Tracey I Sex hormones and pain: the evidence from functional imaging. Curr Pain Headache Rep 2010;14:396–403. 20697845 10.1007/s11916-010-0139-1

[83] Vincent K Warnaby C Stagg CJ Moore J Kennedy S Tracey I Brain imaging reveals that engagement of descending inhibitory pain pathways in healthy women in a low endogenous estradiol state varies with testosterone. PAIN 2013;154:515–24. 23318125 10.1016/j.pain.2012.11.016

[84] Wang Y Wang X Chen W Gupta K Zhu XH Functional MRI BOLD response in sickle mice with hyperalgesia. Blood Cell Mol Dis 2017;65:81–5. 10.1016/j.bcmd.2017.03.005 PMC5521217 28579187

[85] Wells JA Shibata S Fujikawa A Takahashi M Saga T Aoki I Functional MRI of the reserpine-induced putative rat model of fibromyalgia reveals discriminatory patterns of functional augmentation to acute nociceptive stimuli. Sci Rep 2017;7:38325. 28079057 10.1038/srep38325 PMC5228122

[86] Welvaert M Rosseel Y On the definition of signal-to-noise ratio and contrast-to-noise ratio for FMRI data. PLoS One 2013;8:e77089. 24223118 10.1371/journal.pone.0077089 PMC3819355

[87] Williams KA Magnuson M Majeed W LaConte SM Peltier SJ Hu X Keilholz SD Comparison of alpha-chloralose, medetomidine and isoflurane anesthesia for functional connectivity mapping in the rat. Magn Reson Imaging 2010;28:995–1003. 20456892 10.1016/j.mri.2010.03.007 PMC3740561

[88] Wu JJ Lu YC Hua XY Ma SJ Xu JG A longitudinal mapping study on cortical plasticity of peripheral nerve injury treated by direct anastomosis and electroacupuncture in rats. World Neurosurg 2018;114:7. 10.1016/j.wneu.2018.02.173 29524702

[89] Wu R Wang F Yang PF Chen LM High-resolution functional MRI identified distinct global intrinsic functional networks of nociceptive posterior insula and S2 regions in squirrel monkey brain. Neuroimage 2017;155:147–58. 28461059 10.1016/j.neuroimage.2017.04.067 PMC6104393

[90] Zhang S Li T Kobinata H Ikeda E Ota T Kurata J Attenuation of offset analgesia is associated with suppression of descending pain modulatory and reward systems in patients with chronic pain. Mol Pain 2018;14:1744806918767512. 29592786 10.1177/1744806918767512 PMC5882045

